# Evaluating Oxidative Stress in Fibromyalgia: Diagnostic Utility and Its Relationship with Clinical and Psychological Parameters

**DOI:** 10.3390/medicina61071248

**Published:** 2025-07-10

**Authors:** Emine Yıldırım Uslu, Muhammed Fuad Uslu, Sevler Yıldız, Muhammed Fatih Tabara

**Affiliations:** 1 Department of Physical Medicine and Rehabilitation, Elazığ Fethi Sekin City Hospital, 23280 Elazig, Turkey; 2 Department of Internal Medicine, Elazığ Fethi Sekin City Hospital, 23280 Elazig, Turkey; dr.fuslu@gmail.com; 3 Department of Psychiatry, Elazığ Fethi Sekin City Hospital, 23280 Elazig, Turkey; dr_sevler@hotmail.com; 4 Department of Psychiatry, Fırat University Hospital, 23200 Elazig, Turkey; fatihtabara@gmail.com

**Keywords:** fibromyalgia, total antioxidant status, total oxidant status

## Abstract

*Background and Objectives*: This study aimed to evaluate the diagnostic value of oxidative stress parameters in fibromyalgia syndrome (FMS) and to investigate their potential associations with disease severity, functional impairment, anxiety, and depression. *Materials and Methods*: The study included 84 participants, consisting of 42 women diagnosed with fibromyalgia (FM) and 42 healthy female controls. Serum levels of total antioxidant status (TAS), total oxidant status (TOS), and malondialdehyde (MDA) were measured in all participants, and the oxidative stress index (OSI) was calculated. Additionally, the Fibromyalgia Impact Questionnaire (FIQ), Beck Depression Inventory (BDI), and Beck Anxiety Inventory (BAI) were administered to assess mental health and functional status. *Results*: The levels of TOS, TAS, MDA, and the OSI were significantly higher in the fibromyalgia group compared to the control group (*p* < 0.001). The relationship between TAS, TOS, MDA, and OSI levels and BDI, BAI, and FIQ scale scores was investigated in the case group, but no significant associations were identified between oxidative stress markers and psychological or functional scores. When all participants were analyzed as a single group, significant correlations were found between TAS, TOS, MDA, and OSI levels and several biochemical parameters, including CRP, AST, free T4, HbA1c, ferritin, and folic acid. *Conclusions*: Our study adds to the growing body of evidence indicating elevated oxidative stress in female patients with fibromyalgia syndrome (FMS) and suggests that TAS, TOS, MDA, and OSI may serve as potential biomarkers for its diagnosis.

## 1. Introduction

Fibromyalgia syndrome (FMS) is classified as a central sensitization syndrome and is characterized by a range of clinical symptoms, including chronic widespread musculoskeletal pain of unknown origin, fatigue, sleep disturbances, cognitive impairments, and psychiatric manifestations [1]. Predominantly affecting women, FMS reaches its peak prevalence of 2–4% between the ages of 50 and 60 years [2]. Although its etiopathogenesis is not yet fully understood, contributing factors are thought to include genetic predisposition, immune system dysfunction, psychological disorders, sleep disturbances, and neurohormonal imbalances [3,4]. Recent studies have suggested that mitochondrial dysfunction and oxidative stress, which play key roles in the pathogenesis of various diseases, may also be involved in the onset and progression of FMS [3,5].

Oxidative stress is defined as an imbalance between oxidants and antioxidants, resulting in the harmful effects of free radicals. Antioxidants are molecules that mitigate cellular damage by neutralizing these oxidants [6]. Because measuring each oxidant and antioxidant individually is impractical, total oxidant status (TOS) and total antioxidant status (TAS) have been developed as reliable biomarkers for evaluating oxidative stress. TOS reflects the overall oxidant load in the serum and indicates the degree of oxidative stress in the body. In contrast, TAS represents the body’s antioxidant defense capacity. A reduction in TAS levels suggests a weakened antioxidant defense system, potentially contributing to cellular dysfunction and disease progression. The oxidative stress index (OSI), calculated as the ratio of TOS to TAS and expressed as a percentage, offers a more comprehensive assessment of the oxidative balance [7]. Lipids are particularly vulnerable to oxidative damage due to their structural properties. Lipid peroxidation, the reaction between oxidants and lipids, leads to the formation of various oxidation products that can compromise membrane integrity and impair cellular function. Malondialdehyde (MDA), a major byproduct of lipid peroxidation, is widely recognized as a reliable biomarker of lipid oxidative damage. Elevated MDA levels reflect increased lipid peroxidation, which is associated with inflammation, cellular injury, and the development of various diseases. Therefore, simultaneous assessment of TOS, TAS, and MDA levels provides valuable insight into the extent of oxidative stress and its potential role in disease pathogenesis [8].

One of the proposed mechanisms in the etiopathogenesis of fibromyalgia syndrome (FMS) is that oxidative stress impairs tissue oxygenation and causes permanent damage to cellular enzyme systems [5]. A correlation between pro-oxidative processes and increased pain sensitivity in FMS has been demonstrated [9]. Several studies have reported elevated levels of TOS, OSI, and TAS in patients with FMS [10], with oxidative stress and MDA levels being associated with symptom severity [11]. Moreover, increased oxidative damage and reduced antioxidant capacity have been implicated in the amplification of pain [12]. Oxidative stress is also believed to contribute to anxiety and depression, which are commonly observed in individuals with FMS [13]. Oxidative stress is increasingly recognized not only as a contributor to peripheral nociception and central sensitization but also as a key factor in the development of psychiatric symptoms such as anxiety and depression. Elevated oxidative stress has been associated with alterations in neurotransmitter metabolism, neuronal plasticity, and neuroinflammation mechanisms that are closely linked to the affective symptoms commonly observed in fibromyalgia syndrome (FMS) patients [13]. Therefore, assessing oxidative stress alongside standardized psychiatric scales, such as the Beck Depression Inventory (BDI) and Beck Anxiety Inventory (BAI), may provide valuable insights into the multidimensional burden of FMS. The relevance of oxidative stress in female FMS patients may be further influenced by sex-specific hormonal and immunological factors. Estrogen exhibits both antioxidant and pro-oxidant properties depending on its concentration and physiological context, while hormonal fluctuations can modulate pain sensitivity and oxidative balance. Moreover, women tend to have higher rates of stress-related disorders and may demonstrate distinct mitochondrial function and inflammatory responses, which could contribute to a unique oxidative stress profile in female patients with FMS [14]. Although serum TAS, TOS, MDA, and OSI levels are increasingly recognized as indicators of oxidative stress, their association with fibromyalgia syndrome (FMS) severity, anxiety, and depression has not been fully elucidated. This study aims to evaluate whether MDA, TAS, TOS, and OSI levels in patients with FMS can serve as potential biomarkers for diagnosis and disease severity assessment, and to investigate their relationship with anxiety and depression levels.

## 2. Materials and Methods

### 2.1. Participants and Study Desgin

This study was conducted in accordance with the Declaration of Helsinki and received approval from the Ethics Committee of Fırat University Faculty of Medicine (approval date: 21 March 2024, approval number: 23355). Between 1 June 2024, and 31 August 2024, a total of 42 female patients diagnosed with fibromyalgia and 42 healthy female volunteers attending the outpatient clinics of Physical Medicine and Rehabilitation and Internal Medicine at Fethi Sekin City Hospital Hospital were enrolled in the study. Participants were matched by age and sex before being included in the study.

The inclusion criteria for the patient group were females aged 18–60 years who were diagnosed with fibromyalgia syndrome (FMS) according to the 2016 American College of Rheumatology (ACR) criteria [15] and had no prior treatment for FMS. Exclusion criteria included advanced cardiovascular, endocrine, metabolic, renal, or hepatic diseases; a history of alcohol or substance abuse or dependence; neurological disorders; inflammatory diseases; and malignancy. For the control group, inclusion criteria comprised females aged 18–60 years who attended the hospital for routine annual check-ups and had no systemic or psychiatric complaints. Exclusion criteria for controls included a history of psychiatric treatment, alcohol or substance abuse within the past six months, and a body mass index (BMI) greater than 30. All participants underwent evaluation by specialists in physical medicine and rehabilitation and/or internal medicine prior to referral to a psychiatrist. During the initial assessment, participants completed the Sociodemographic and Clinical Data Form as well as the Fibromyalgia Impact Questionnaire (FIQ). Structured interviews lasting at least 30 min were conducted by a psychiatrist according to the Diagnostic and Statistical Manual of Mental Disorders, Fifth Edition (DSM-5) criteria. Additionally, the Beck Anxiety Inventory (BAI) and Beck Depression Inventory (BDI) were administered.

### 2.2. Clinical Variables and Blood Biomarker Analysis

To minimize the effects of circadian variation on oxidative stress markers, blood samples were collected from all participants between 8:00 and 9:00 am. All participants were instructed to fast for at least 8 h prior to the blood draw, and compliance with the fast was confirmed verbally upon arrival. Venipuncture was performed using the standard aseptic technique, and approximately 10 mL of venous blood was drawn from the antecubital vein. Following collection, the blood samples were immediately placed in the correct tubes for biochemical and hematological analyses. Samples designated for oxidative stress analysis (TAS, TOS, and MDA) were allowed to clot at room temperature for 20–30 min, after which they were centrifuged at 3000 rpm for 10 min. The resulting serum was carefully divided into labeled Eppendorf tubes and stored at −70 °C until analysis, to prevent degradation of the analytes. All samples were processed within one hour of collection to ensure sample integrity, and oxidative stress measurements were conducted in batches within two months of collection. Laboratory personnel performing the assays were blinded to the group allocation of the samples to minimize bias. Routine biochemical analyses were performed using an automated analyzer (Beckman AU 5800, Brea, CA, USA). For routine hemogram tests, 3 mL of EDTA-anticoagulated whole blood was used, analyzed by an automated hematology analyzer (Beckman Coulter DXH 800, Brea, CA, USA).

TAS levels were measured using commercially available kits (Relassay, Turkey). The new automated method is based on the bleaching of the characteristic color of an ABTS (2,2′-Azino-bis(3-ethylbenzothiazoline-6-sulfonic acid)) radical cation by antioxidants. This test offers excellent precision with values below 3%. The results were expressed in mmol Trolox equivalent/L (Erel O. A novel automated direct measurement method for total antioxidant capacity using a new generation, more stable ABTS radical cation [8].) (Relassay, Turkey).

TOS levels were measured using commercially available kits (Relassay, Turkey). In this new method, the oxidants in the sample oxidized the ferrous ion-o-dianisidine complex to ferric ion. The oxidation reaction was enhanced by the glycerol molecules present in the reaction medium. The ferric ion then formed a colored complex with xylenol orange in an acidic medium. The color intensity, which could be measured spectrophotometrically, was directly related to the total amount of oxidant molecules in the sample. The assay was calibrated using hydrogen peroxide, and the results were expressed as micromolar hydrogen peroxide equivalent per liter (μmol H_2_O_2_ equivalent/L) (Erel O. A new automated colorimetric method for measuring total oxidant status [16].) (Relassay, Turkey).

The ratio of TOS to TAS was considered the oxidative stress index (OSI). To calculate the OSI, the resulting unit of TAS was converted to μmol/L, and the OSI value was determined using the following formula:

OSI (arbitrary unit) = TOS (μmol H_2_O_2_ equivalent/L)/TAS (μmol Trolox equivalent/L).

Biochemical assays were conducted by laboratory personnel blinded to the participants’ case/control status. Psychiatric interviews, however, were performed by a clinician aware of group assignments due to the necessity of contextual judgment during structured assessments.

### 2.3. Scales Used in the Study

Sociodemographic and Clinical Data Form: This semi-structured form, prepared by us, includes clinical data such as place of residence, duration of illness, and presence of comorbidities.

Beck Anxiety Inventory (BAI): The Beck Anxiety Inventory (BAI) is a questionnaire designed to assess the severity of anxiety symptoms experienced by participants. Higher total scores indicate greater levels of anxiety. The inventory was developed by Beck et al. [17], and its Turkish validity and reliability were established by Ulusoy et al. [18]. Anxiety is among the most common psychological symptoms in patients with fibromyalgia and has been suggested to be associated with oxidative stress. The BAI is a widely used and validated instrument for the objective evaluation of anxiety levels. Assessing anxiety symptoms in relation to oxidative stress markers may provide valuable insights into the psychological effects of oxidative stress in fibromyalgia patients.

Beck Depression Inventory (BDI): The Beck Depression Inventory (BDI) is a questionnaire that assesses the frequency of depressive symptoms experienced by participants. Higher total scores indicate greater severity of depression. The inventory was developed by Beck et al. [19], and its Turkish validity and reliability have been established [20]. Depression is a common psychiatric condition among fibromyalgia patients and has been associated with elevated oxidative stress levels in previous studies. The BDI is a well-established instrument for evaluating the severity of depressive symptoms. In this study, the BDI was used to explore the potential relationship between oxidative stress and depression in fibromyalgia patients. Understanding this association may underscore the significance of psychological factors in the management of fibromyalgia.

Fibromyalgia Impact Questionnaire (FIQ): The Fibromyalgia Impact Questionnaire (FIQ) was developed to assess functional status in patients with fibromyalgia syndrome (FMS). Its validity and reliability were established by Sarmer et al. [21]. Since oxidative stress may influence not only biochemical parameters but also disease severity at the functional level, the FIQ was included to evaluate the overall impact of fibromyalgia on daily living. The questionnaire assesses multiple dimensions, including physical functioning, pain severity, fatigue, morning stiffness, and psychological well-being. Utilizing the FIQ enables a more comprehensive analysis of the potential relationship between oxidative stress, mental health, and functional impairment in patients with fibromyalgia.

By incorporating BAI and BDI, this study aimed to evaluate the psychological dimensions of oxidative stress, while FIQ provided insight into its potential effects on functional status. This combination of scales enables a holistic understanding of the impact of oxidative stress on both mental health and quality of life in fibromyalgia patients.

### 2.4. Statistical Analysis

Analyses were performed using SPSS (Statistical Package for the Social Sciences; SPSS Inc., Chicago, IL, USA) version 22 software package. Categorical variables such as marital status, educational status, alcohol use, and smoking were compared using the Pearson Chi-square test. Descriptive statistics were presented using frequencies (*n*) and percentages (%) for categorical variables and mean and standard deviation, median, and interquartile range (25th–75th percentile values) for continuous variables. The Kolmogorov–Smirnov test, kurtosis-distribution, and histogram graphs were used to evaluate the conformity of continuous variables to normal distribution. The Mann–Whitney U test was used to compare the age, platelet, lymphocyte, neutrophil, monocyte, sedimentation, CRP, AST, ALT, urea, insulin, HOMA-IR, vitamin D, vitamin B12, folic acid, TSH, ferritin, TOS, and OSI values of the groups. BMI, systolic and diastolic TA, WBC, hemoglobin, glucose, creatine, free T4, HbA1c, TAS, and MDA values were compared with the Student’s t-test. The relationship between continuous numerical variables was investigated by Spearman’s correlation test. In addition, the relationship between BDI, BAI, and FIQ scales and TAS, TOS, MDA, and OSI in the case group was also evaluated by the Spearman correlation test. Whether age, BMI, TAS, TOS, MDA, OSI, HbA1c, HOMA-IR, vitamin D, vitamin B12, folic acid, and free T4 values predicted FIQ scores were analyzed in a multiple linear regression model. The Benjamini–Hochberg procedure was applied for multiple comparisons. A significance level of *p* < 0.05 was accepted for all analyses. A post-hoc power analysis based on TAS values indicated a statistical power of 100% (Cohen’s d = 4.33, α = 0.05, *n* = 42 per group).

## 3. Results

The study included 84 participants, comprising 42 women in the case group and 42 women in the control group. The median age of the case group was 40.5 years (interquartile range [IQR] = 30.75–46.75), while the median age of the control group was 30 years (IQR = 27–45.5) (*p* > 0.05). In the case group, 36 (85.7%) participants were single, compared to 25 (59.5%) in the control group (*p* = 0.007). The use of alcohol and cigarettes was similar between the groups, with the majority reporting no alcohol consumption (*p* > 0.05). There were no significant differences between the groups in terms of educational status (*p* > 0.05). Comparisons of the sociodemographic characteristics of the groups are presented in Table 1.

**Figure 1:**
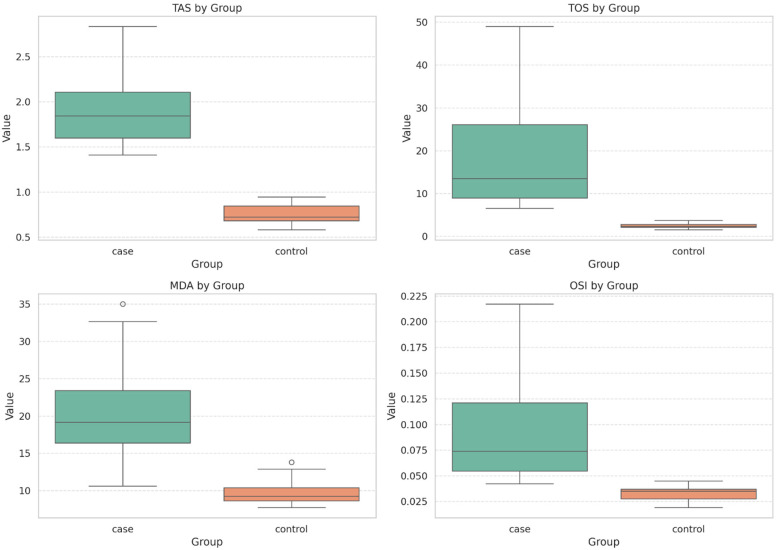
Comparison of TAS, TOS, MDA, and ODI Parameters Between Groups. Bar plots represent group means ± standard deviations. TAS: Total Antioxidant Status; TOS: Total Oxidant Status; MDA: Malondialdehyde; OSI: Oxidative Stress Index. Statistical comparisons were made using Student’s t-test for normally distributed variables (TAS and MDA) and the Mann–Whitney U test for non-normally distributed variables (TOS and OSI). *p*  <  0.001 for all comparisons.

The median TOS level in the case group was 13.5 (IQR = 8.85–27.64), while the median TOS level in the control group was 2.37 (IQR = 2.10–2.82). The TOS levels in the case group were significantly higher than those in the control group (*p* < 0.001, Cohen’s *d* = 3.38, Eta squared (*η^2^*) = 0.74). The median OSI value in the case group was 0.073 (IQR = 0.053–0.128), whereas the median OSI value in the control group was 0.035 (IQR = 0.027–0.036) (*p* < 0.001, Cohen’s *d* = 3.34, Eta squared (*η^2^*) = 0.74). In terms of TAS values, the mean TAS value in the case group (1.92 ± 0.37) was significantly higher than that in the control group (0.75 ± 0.09) (*p* < 0.001, Cohen’s *d* = 4.34, r = 0.90). Similarly, the mean MDA value in the case group (20.70 ± 6.14) was higher than that in the control group (9.64 ± 1.46) (*p* < 0.001, Cohen’s *d* = 2.48, r = 0.78). Comparative analysis of TAS, TOS, MDA, and OSI parameters between the fibromyalgia patients and the healthy control group is shown in the figure (Figure 1).

A post-hoc power analysis was conducted based on TAS values to evaluate the adequacy of the sample size. Using a two-sample t-test with α = 0.05, an effect size (Cohen’s d) of 4.33, and equal group sizes (*n* = 42), the calculated statistical power was found to be 100%.

BMI, arterial blood pressure, hemogram, and biochemical values were compared between the groups. Both systolic and diastolic arterial blood pressure levels in the case group were significantly lower than those in the control group. Additionally, BMI, CRP, ferritin, AST, HbA1c, and folic acid levels were significantly higher in the case group compared to the control group. In terms of other hemogram and biochemical parameters, the groups were similar. A comparison of the blood parameters between the groups is presented in Table 2.

When all participants were evaluated together, significant correlations were found between TAS, TOS, MDA, and OSI levels and age, BMI, arterial blood pressure, CRP, AST, free T4, HbA1c, ferritin, and folic acid levels. The test values for these correlations are presented in Table 3.

Additionally, the relationship between TAS, TOS, MDA, and OSI levels and BDI, BAI, and FIQ scale scores was investigated solely in the case group, but no statistically significant correlations were found. Test values for the comparisons are given in Table 4.

In the case group, a linear regression analysis was performed to assess whether age, BMI, TAS, TOS, MDA, OSI, HbA1c, HOMA-IR, vitamin D, vitamin B12, folic acid, and free T4 levels could predict FIQ scores. The results indicated that none of these variables had a statistically significant effect (*p* > 0.05). The data are presented in Table 5.

## 4. Discussion

This study aimed to evaluate whether TAS, TOS, MDA, and OSI levels could serve as biomarkers for the diagnosis and severity assessment of fibromyalgia syndrome (FMS). We found that TOS, TAS, OSI, and MDA levels were significantly elevated in women with FMS compared to the control group. However, no significant correlations were observed between these oxidative stress markers and scores on the Beck Depression Inventory (BDI), Beck Anxiety Inventory (BAI), or Fibromyalgia Impact Questionnaire (FIQ). Additionally, the levels of CRP, ferritin, AST, HbA1c, and folic acid were higher in FMS patients than in controls.

There is no definitive objective test for confirming the diagnosis of fibromyalgia syndrome (FMS). Blood biomarkers have been proposed as potential objective measures, and numerous studies have investigated this topic. A meta-analysis of blood biomarker studies in fibromyalgia syndrome (FMS) demonstrated increased levels of pro-inflammatory cytokines and decreased concentrations of the anti-inflammatory cytokine interleukin-1β in patients compared to healthy controls [22]. However, the same meta-analysis concluded that none of these biomarkers were specific to FMS. Besides inflammation, other factors such as microinflammation, intestinal microbiota, metabolic syndrome, and oxidative stress have also been explored as potential biomarkers for FMS [23]. Oxidative stress has received increasing attention in fibromyalgia syndrome (FMS) due to growing evidence of its involvement in the disease. It is considered a key factor in the pathogenesis of FMS. Decreased levels of coenzyme Q10 in FMS are believed to reduce mitochondrial membrane potential, leading to increased superoxide production and synthesis of lipid peroxidation products. Brain tissue is particularly vulnerable to oxidative stress because of its high lipid content. Oxidative stress is thought to contribute to nociception and hyperalgesia by influencing both peripheral and central sensitization [24]. Bozkurt et al. [25] reported elevated TOS and OSI levels in FMS patients compared to controls. Similarly, Çetinkaya et al. [26] found that FMS patients had increased TOS and OSI levels and decreased TAS levels compared to controls. In our study, we found that TOS, OSI, and TAS levels were all higher in patients with FMS than in the control group. However, many previous studies have reported decreased TAS levels in this population. This discrepancy may be due to several factors, such as methodological differences, variations in assay sensitivity, or compensatory upregulation of antioxidant defenses in response to increased oxidative stress. Additionally, regional or nutritional differences within the study population may have affected systemic antioxidant capacity. Bagis et al. [11] reported that patients with FMS had elevated MDA levels compared to the control group. Consistent with these findings, our study also observed increased MDA levels in patients with FMS. These results support the idea that oxidative stress is higher in patients with FMS than in healthy people and suggest that TAS, TOS, OSI, and MDA could be used as diagnostic markers for FMS. However, we found no significant correlations between these levels and Fibromyalgia Impact Questionnaire (FIQ) scores. In contrast, Tel Adıgüzel et al. [27] reported a positive correlation between TOS and OSI levels, as well as FIQ scores. Additionally, several studies have demonstrated correlations between MDA levels and fibromyalgia severity [28,29]. Altındağ et al. [30] observed a significant negative correlation between total antioxidant capacity and pain severity. The discrepancy between our results and those reported in the literature may be due to the small sample size and the fact that the study only included females.

Psychiatric comorbidity is highly prevalent among patients with fibromyalgia syndrome (FMS), with frequent associations reported between FMS and depression and anxiety [31]. According to the results of the standardized scale in our study, the levels of depression in the patient group were classified as moderate, while the levels of anxiety were classified as severe. Both depression and anxiety have been shown to be associated with oxidative stress [32]. To our knowledge, no prior studies have specifically examined the relationship between oxidative stress markers and the depression and anxiety symptoms commonly observed in FMS. In the present study, however, we found no significant correlations between these markers and scores on the depression and anxiety scales.

The absence of a correlation between oxidative biomarkers and symptom severity suggests that oxidative stress may represent only one of several overlapping mechanisms involved in FMS. Our findings are consistent with the recent literature emphasizing the interconnected roles of oxidative stress, psychological symptoms, and physical function in this syndrome. For example, interventions such as physical activity and manual therapy have been shown to affect physiological and emotional outcomes in women with FMS, highlighting the importance of interpreting oxidative biomarkers within a comprehensive biopsychosocial framework [33,34].

In our study, the patient group had significantly lower blood pressure than the control group. Previous research has suggested that autonomic nervous system dysfunction may be involved in fibromyalgia syndrome (FMS) [35]. Katz et al. proposed that vasomotor dysregulation in FMS could lead to muscle ischemia and contribute to the development of pain [36]. Similarly, Paso et al. [37] reported lower diastolic blood pressure in FMS patients, which is consistent with our findings. Overall, our results are consistent with the existing literature on this topic.

In our study, C-reactive protein (CRP) levels were significantly higher in the FMS group compared to controls, indicating an elevated inflammatory response associated with the condition. It is believed that inflammatory mediators, such as cytokines, contribute to pain by disrupting nociceptive signaling [38]. This finding is consistent with previous research demonstrating a link between inflammation and pain in FMS patients. For example, one study examined erythrocyte sedimentation rate (ESR), CRP, interleukin-6 (IL-6), and interleukin-8 (IL-8) levels. While ESR, IL-6, and IL-8 levels did not differ significantly between FMS patients and healthy controls, CRP levels were notably higher in FMS patients, which supports our findings [39]. Understanding the interplay between oxidative stress and inflammation is key to grasping the pathophysiology of fibromyalgia. Oxidative stress promotes the production of reactive oxygen species (ROS), which stimulate the release of pro-inflammatory cytokines and perpetuate inflammation. This creates a vicious cycle in which elevated oxidative stress exacerbates inflammation, contributing to the persistent pain and fatigue experienced by patients with fibromyalgia. Furthermore, the intensified inflammatory response may enhance pain sensitivity by amplifying nociceptive signaling, thus perpetuating fibromyalgia symptoms. Recent studies have also indicated that oxidative stress markers correlate with pain severity and other clinical manifestations of fibromyalgia, suggesting that targeting oxidative stress could be an effective therapeutic strategy. Antioxidant therapies have shown promise in reducing inflammation and improving clinical outcomes, underlining the importance of a multifaceted treatment approach that addresses both oxidative stress and inflammation. In summary, our findings emphasize the importance of investigating the complex relationship between oxidative stress and inflammatory markers in fibromyalgia. A better understanding of this relationship advances our insights into disease pathophysiology and paves the way for novel therapeutic interventions aimed at relieving symptoms and enhancing patients’ quality of life.

In our study, HbA1c levels were significantly higher in patients with FMS than in the control group. It is reported that the prevalence of metabolic syndrome and diabetes mellitus is higher in patients with FMS than in the general population [40,41]. Several studies have also found elevated HbA1c levels in FMS patients compared to the control group, which is consistent with our findings [42]. These results suggest that HbA1c could be used as a marker to distinguish between FMS and other conditions, even when values remain within the normal range. Additionally, ferritin and folate levels were higher in the FMS group than in healthy controls. Although low levels of ferritin, vitamin B12, vitamin D, and folate are commonly reported in association with FMS [43,44], we hypothesize that the elevated ferritin and folate levels observed in our cohort may be due to frequent vitamin supplementation prescribed to patients with chronic pain by other specialists

The limitations of our study include the fact that we only included female patients. This may restrict the generalizability of our findings to the broader fibromyalgia population, including men. Additionally, this was a single-center, cross-sectional study with a relatively small sample size. While no significant age difference was observed between the groups, the higher median age of the FMS group could have influenced the oxidative stress parameters, given that age-related metabolic and inflammatory changes may act as confounding factors. Furthermore, data on influential variables such as dietary habits, use of antioxidants or vitamin supplements, and physical activity levels were not collected, which may have impacted oxidative biomarker measurements. Due to the cross-sectional design, it is unclear whether oxidative stress contributes to the development of pain and psychiatric symptoms in FMS or is a consequence of chronic symptom burden. Future research should aim to include more diverse populations in terms of gender, age, and geographic location and employ longitudinal designs to clarify causal relationships and enhance external validity.

## 5. Conclusions

This study observed significantly higher levels of TAS, TOS, MDA, and OSI in female fibromyalgia patients than in healthy controls, suggesting a potential imbalance in oxidative stress. While these results imply that oxidative stress markers could contribute to the biological profile of FMS, they should not yet be considered definitive diagnostic tools. Notably, no significant associations were found between oxidative markers and clinical severity or psychiatric scores, which highlights the complex and multifactorial nature of the syndrome. While our results support the hypothesis that oxidative imbalance may play a role in the pathophysiology of FMS, longitudinal and prospective studies are needed to establish causality. Future research should also include male participants, control for lifestyle and comorbid factors, and incorporate repeated biomarker assessments to improve clinical applicability.

## Figures and Tables

**Table 1:** Comparison of sociodemographic data of the groups.

		Case Group (*n* = 42) *n* (%)	Control Group (*n* = 42) *n* (%)	*p* Value
Age (Median ± IQR)		40.50 (30.75–46.75)	30 (27–45.5)	0.082 ^a^
Sex	Woman	42 (%100)	42 (%100)	
BDI (Median ± IQR)		20.50 (12.75–27.50)	-	-
BAI (Median ± IQR)		31 (16–38.5)	-	-
FIQ (Median ± IQR)		57.66 (44.99–62.66)	-	-
Marital status				0.007 ^b^
	Single	36 (%85.7)	25 (%59.5)	
	Married	6 (%14.3)	17 (%40.5)	
Education status				0.890 ^b^
	Primary education	17 (%40.5)	16 (%38.1)	
	High School	11 (%26.2)	13 (%31)	
	University	14 (%33.3)	13 (%31)	
Alcohol use				1.000 ^b^
	No	37 (%88.1)	37 (%88.1)	
	Yes	5 (%11.9)	5 (%11.9)	
Cigarette smoking				1.000 ^b^
	No	31 (%73.8)	31 (%73.8)	
	Yes	11 (%26.2)	11 (%26.2)	

^a^ Mann–Whitney U test; ^b^ Chi-square test; BMI: Body mass index; IQR: Interquartile Range; BDI: Beck Depression Inventory; BAI: Beck Anxiety Inventory; FIQ: Fibromyalgia Impact Questionnaire.

**Table 2:** Comparison of blood parameters of the groups.

	Case Group (*n* = 42) (Median ± IQR)/(Mean ± Sd)	Control Group (*n* = 42) (Median ± IQR)/(Mean ± Sd)	*p* Value
OSI	0.073 (0.053–0.128)	0.035 (0.027–0.036)	<0.001 ^a^
TOS (µmol/L)	13.5 (8.85–27.64)	2.37 (2.10–2.82)	<0.001 ^a^
TAS (mmol/L)	1.92 ± 0.37	0.75 ± 0.09	<0.001 ^b^
MDA (nmol/mL)	20.70 ± 6.14	9.64 ± 1.46	<0.001 ^b^
BMI (kg/m^2^)	26.05 ± 3.47	24.88 ± 2.80	0.095 ^b^
Systolic BP (mmHg)	114.73 ± 20.79	124.4 ± 11.65	0.011 ^b^
Diastolic BP (mmHg)	75.71 ± 13.59	80.43 ± 6.68	0.048 ^b^
WBC (10^9^/L)	6.36 ± 1.45	6.47 ± 1.41	0.727 ^b^
Hemoglobin (g/dL)	13.18 ± 1.34	13.12 ± 1.13	0.814 ^b^
Platelets (10^9^/L)	292 (243.25–344.25)	271 (237.25–331)	0.591 ^a^
Neutrophil (10^9^/L)	3.28 (2.77–4.48)	3.73 (2.9–4.92)	0.488 ^a^
Lymphocyte (10^9^/L)	1.92 (1.47–2.50)	1.89 (1.57–2.39)	0.954 ^a^
Monocyte (10^9^/L)	0.51 (0.41–0.58)	0.45 (0.35–0.55)	0.153 ^a^
Sedimentation (mm/h)	9 (5–16.5)	7 (5–11)	0.268 ^a^
CRP (mg/L)	3.36 (1.29–5.37)	1.81 (0.92–3.19)	0.013 ^a^
Ferritin (µg/L)	17 (7.5–34.25)	8 (4.75–17)	0.011 ^a^
Glucose (mg/dL)	88.4 ± 10.84	87.74 ± 5.49	0.723 ^b^
Insulin	8.93 (6.06–12.94)	7.67 (5.05–10.94)	0.279 ^a^
HOMA-IR	1.86 (1.3–2.7)	1.62 (1.03–2.28)	0.316 ^a^
HbA1c	5.55 ± 0.27	5.33 ± 0.33	0.002 ^b^
AST (U/L)	18 (16–23.25)	17 (15–19)	0.018 ^a^
ALT (U/L)	14 (11.75–20.25)	13 (11–21)	0.628 ^a^
Urea (mg/dL)	23.5 (20–30)	22 (18.75–28.5)	0.563 ^a^
Creatine (mg/dL)	0.59 ± 0.08	0.63 ± 0.09	0.067 ^b^
Vitamin D (µg/L)	15.5 (10.75–24)	14 (11.6–17.5)	0.418 ^a^
VitaminB12 (ng/L)	179 (147.75–281.25)	166 (132–222)	0.232 ^a^
Folic acid (µg/L)	8.16 (6.16–10.75)	6.51 (5.08–7.6)	<0.001 ^a^
TSH (mIU/L)	1.54 (1.23–2.46)	1.74 (1.16–2.57)	0.865 ^a^
Free T4 (ng/dL)	0.84 ± 0.12	0.82 ± 0.10	0.295 ^b^

^a^ Mann–Whitney U test; ^b^ Student’s *t*-test; IQR: Interquartile Range; Sd: Standard deviation; OSI: Oxidative stress index; TOS: Total Oxidant Status; TAS: Total Antioxidant Status; MDA: Malondialdehyde; BP: Blood Pressure; WBC: White Blood Cell; CRP: C-reactive protein; HOMA-IR: Homeostasis model assessment of insulin resistance; HbA1c: Glycosylated hemoglobin; ALT: Alanine aminotransferase; AST: Aspartate aminotransferase.

**Table 3:** Correlations between participants’ parameters.

		TAS	TOS	MDA	OSI
Age	r *p*	0.190 0.083	0.227 **0.038**	0.251 **0.021**	0.233 **0.033**
BMI	r *p*	0.197 0.073	0.196 0.074	0.163 0.138	0.372 **0.042**
Systolic BP	r *p*	−0.425 **<0.001**	−0.315 **0.004**	−0.293 **0.007**	−0.277 **0.011**
Diastolic BP	r *p*	−0.340 **0.002**	−0.271 **0.013**	−0.251 **0.022**	−0.252 **0.021**
CRP	r *p*	0.187 0.088	0.206 0.060	0.240 **0.028**	0.211 0.054
AST	r *p*	0.273 **0.012**	0.216 **0.049**	0.289 **0.008**	0.180 0.101
Free T4	r *p*	0.124 0.263	0.213 0.052	0.089 0.420	0.244 **0.025**
HbA1c	r *p*	0.297 **0.006**	0.333 **0.002**	0.361 **0.001**	0.346 **0.001**
Ferritin	r *p*	0.278 **0.010**	0.326 **0.002**	0.232 **0.033**	0.296 **0.006**
Folic acid	r *p*	0.367 **0.001**	0.358 **0.001**	0.294 **0.007**	0.352 **0.001**
Spearman correlation test Comparisons with statistically significant differences are indicated in **bold**.					

BMI: Body Mass Index; BP: Blood Pressure; CRP: C-reactive protein; AST: Aspartate aminotransferase; HbA1c: Glycosylated hemoglobin.

**Table 4:** The correlation between patients’ scale scores and inflammation markers.

		TAS	TOS	MDA	OSI
BDI	r *p*	0.193 0.221	0.157 0.321	−0.025 0.873	0.131 0.407
BAI	r *p*	−0.035 0.826	−0.173 0.273	−0.178 0.260	−0.253 0.107
FIQ	r *p*	−0.174 0.271	−0.174 0.271	0.075 0.636	−0.181 0.251

Spearman correlation test; BDI: Beck Depression Inventory; BAI: Beck Anxiety Inventory; FIQ: Fibromyalgia Impact Questionnaire; OSI: Oxidative stress index; TOS: Total Oxidant Status; TAS: Total Antioxidant Status; MDA: Malondialdehyde.

**Table 5:** Multiple linear regression analysis of FIQ scores.

Variable	Coefficient (β)	Standard Error	t-Statistic	*p*-Value	95% Confidence Interval	Adjusted *p* *
Intercept	142.6033	57.748	2.469	0.020	24.496–260.711	
Age	0.5223	0.277	1.887	0.069	−0.044–1.088	0.276
BMI	−0.0843	0.807	−0.104	0.918	−1.734–1.566	0.993
TAS	−2.4807	23.177	0.107	0.916	−49.883–44.922	0.993
TOS	1.1087	1.684	0.658	0.516	−2.336–4.553	0.810
MDA	0.0028	0.367	0.008	0.994	−0.747–0.753	0.993
OSI	−407.7107	349.317	−1.167	0.253	−1122.145–306.723	0.606
HbA1c	−18.0923	9.462	1.912	0.066	−37.443–1.259	0.276
HOMA-IR	−0.2069	0.866	−0.239	0.813	−1.977–1.563	0.993
Vitamin D	−0.2633	0.325	−0.810	0.424	−0.928–0.401	0.810
Vitamin B12	0.0076	0.012	0.620	0.540	−0.017–0.032	0.810
Folic acid	−0.9866	0.477	−2.068	0.048	−1.962–−0.011	0.276
Free T4	31.3476	22.047	1.422	0.166	−13.743–76.439	0.497

* Benjamini–Hochberg FDR correction; BMI: Body Mass Index; OSI: Oxidative stress index; TOS: Total Oxidant Status; TAS: Total Antioxidant Status; MDA: Malondialdehyde; HOMA-IR: Homeostasis model assessment of insulin resistance; HbA1c: Glycosylated hemoglobin.

## Data Availability

The original contributions presented in this study are included in the article. Further inquiries can be directed to the corresponding author(s).

## Abbreviations

The following abbreviations are used in this manuscript:

FMS	Fibromyalgia Syndrome
TAS	Total Antioxidant Status
TOS	Total Oxidant Status
MDA	Malondialdehyde
